# Electrolytes supplementation can decrease the risk of nephrotoxicity in patients with solid tumors undergoing chemotherapy with cisplatin

**DOI:** 10.1186/s40360-020-00448-9

**Published:** 2020-09-23

**Authors:** Omary M. S. Minzi, Tatu E. Lyimo, Francis F. Furia, Alphonce I. Marealle, Manase Kilonzi, George M. Bwire, Christina Malichewe

**Affiliations:** 1 Department of Clinical Pharmacy and Pharmacology, School of Pharmacy, Muhimbili University of Health and Allied Sciences, P. O. Box 65013, Dar es Salaam, Tanzania; 2 Department of Paediatrics and Child Health, School of Medicine, Muhimbili University of Health and Allied Sciences, P. O. Box 65001, Dar es Salaam, Tanzania; 3 Department of Pharmaceutical Microbiology, School of Pharmacy, Muhimbili University of Health and Allied Sciences, P. O. Box 65013, Dar es Salaam, Tanzania; 4 Department of Radiology, MUHAS, P.O. Box 65000, Dar es Salaam, Tanzania

**Keywords:** Triple electrolyte supplementation, Cancer, Cisplatin, nephrotoxicity, Serum creatinine, normal saline

## Abstract

**Background:**

Cisplatin is an important drug in the treatment of various Cancers. However, this drug causes nephrotoxicity that is linked to electrolyte derangement. The aim of this study was to evaluate the effect of electrolyte supplementation in reducing kidney injury in patients receiving cisplatin-based regimen.

**Methods:**

This was non-randomized interventional study conducted at Ocean Road Cancer Institute (ORCI) among patients with confirmed solid tumors. Patients who received cisplatin-based chemotherapy at a dose of ≥50 mg with intravenous normal saline supplemented with Magnesium, Calcium and Potassium (triple electrolyte supplementation) were compared with those who received cisplatin-based chemotherapy with normal saline alone. The patients were followed up for 4 weeks and serum creatinine was measured at every visit. Nephrotoxicity was defined as serum creatinine elevation > 1.5 times that at baseline.

**Results:**

A total of 99 patients were recruited, whereby 49 patients (49.5%) received electrolyte supplementation (treatment group) and 50 patients (51.5%) did not receive electrolyte supplementation (control group). The incidence risk of nephrotoxicity was 20.41% (*n* = 10) in the treatment group and 54% (*n* = 27) in the control group. Patients in the control group were 2.6 times more likely to experience nephrotoxicity as compared to treatment group [Relative Risks (RR); 2.6, 95%CI; 1.5–4.9, *P* < 0.0001]. The most common malignancy was cervical cancer, *n* = 43 (87.8%) in treatment group and *n* = 45 (90.0%) in the control group (*P* = 0.590). The Kaplan-Meier analysis and the log-rank test revealed that electrolytes supplementation was associated with extended survival with less nephrotoxicity incidences [*P* = 0.0004; Hazard ratio (HR) 0.3149; 95% CI 0.165 to 0.6011].

**Conclusions:**

Electrolytes supplementation decreases the risk of nephrotoxicity after chemotherapy with cisplatin. A randomized controlled trial with a larger sample size is recommended to evaluate the robustness of these findings.

## Introduction

Cancer is currently becoming a health problem concern in several less developed and economically transitioning countries. While the incidence and mortality rates for most cancers in the United States and many other western countries are decreasing, the trend in several less developed and economically transitioning countries is the opposite because of adoption of unhealthy western lifestyles such as smoking, physical inactivity, and consumption of calorie-dense food [1, 2]. Most developing countries also continue to be disproportionately affected by cancers related to infectious agents, such as cervical, liver, and stomach cancers. The proportion of new cancer cases diagnosed in less developed countries is projected to increase from about 56% of the world total in 2008 to more than 60% in 2030 [1]. As opposed to European countries, lack of access to good medical services such as lack organized screening and human papillomavirus (HPV) vaccination programmes as well as sudden increase in population play major role in the increased number of cancer cases in less developed African countries [3].

To contain the trends of increased morbidity and mortality in these countries, there is an urgent need for instituting preventive strategies such as lifestyle changes and also educating the population to timely turn to health facilities for early detection, diagnosis, and treatment.

There are three modalities of treating solid tumors; surgery, radiotherapy, and chemotherapy. Cisplatin, a platinum-based antineoplastic agent and a well-known chemotherapeutic drug is the cornerstone for the treatment of many malignancies including bladder, head and neck, lung, ovarian, and testicular cancers. It is effective against various types of cancers, including carcinomas, germ cell tumors, lymphomas, and sarcomas [4]. Cisplatin is used as a single agent or in combination with other drugs and acts by cross-linking the purine bases on the DNA; interfering with DNA repair mechanisms, causing DNA damage, and subsequently inducing apoptosis in cancer cells. However, because of drug resistance and numerous undesirable side effects such as severe kidney problems, allergic reactions, decrease immunity to infections, gastrointestinal disorders, hemorrhage, and hearing loss especially in younger patients, other platinum-containing anti-cancer drugs such as carboplatin, oxaliplatin, and others are used [5, 6]. Combining cisplatin with other drugs is a good strategy for overcoming its resistance and reducing toxicity. Cisplatin and its derivatives have debilitating side effects in normal tissues and induce ototoxicity, neurotoxicity, and nephrotoxicity. In kidneys, cisplatin preferentially accumulates in renal tubular cells causing tubular cell injury and death, resulting in acute kidney injury (AKI) [5, 6].

Nephrotoxicity is the primary dose-limiting toxicity, and various hydration regimens and supplementation strategies are used to prevent cisplatin-induced kidney injury [5–8]. Acute nephrotoxicity has been linked to electrolyte disturbances and dehydration, and several studies have shown that the use of cisplatin is associated with the derangement of magnesium, potassium, and calcium [9–13]. Adequate hydration before, during and after cisplatin therapy with saline and simultaneous administration of mannitol significantly reduce cisplatin nephrotoxicity and this strategy has been accepted as the standard of care in some clinical settings [14].

To date, several studies have investigated strategies for prevention of cisplatin-induced renal toxicity. Some studies have shown that hydration and supplementation with either magnesium or calcium or both have a renal protective effect in patients receiving cisplatin-based chemotherapy [15, 16]. As free cisplatin causes renal toxicity, shortening the free cisplatin and renal tubular contact time is important to reduce its nephrotoxicity [16]. Recently Crona et al. wrote a systematic review in which various renoprotective strategies in patients receiving cisplatin-based therapy were described [5]. In 2012, Arunkumar et al. was also able to show that five cycles of cisplatin-based chemotherapy resulted in hypomagnesia, hypocalcemia, hypophosphatemia, hypokalemia, and increased serum creatinine and Blood Urea Nitrogen (BUN) levels suggesting the importance of electrolyte supplementation of the depleted ions [9].

Based on the lack of guidance regarding cisplatin hydration, multiple hydration protocols exist among different health systems. In this study, we report the protective effect of triple electrolyte supplementation with potassium, magnesium, and calcium among patients with solid tumors who were undergoing cisplatin-based chemotherapy at ocean Road Cancer Institute in Dar Es Salaam, Tanzania.

## Methods

### Study design

This was non-randomized interventional study that was conducted to evaluate reno-protective effects of intravenous triple electrolytes supplementation in reducing the incidenceof nephrotoxicity among chemotherapy-naive cancer patients following the course of standard cisplatin-based chemotherapy at Ocean Road Cancer Institute (ORCI).

A cohort of patients who received cisplatin-based chemotherapy was studied and patients who received the drug plus electrolyte supplements and normal saline were compared with those who received the drug and normal saline alone. The triple electrolyte supplementation used contained Potassium chloride (KCl) (1.5 g), Magnesium sulfate (1 g) and calcium gluconate (1 g).

### Study area

The study was conducted at ORCI, Dar es Salaam-Tanzania between January 2019 and June 2019. ORCI is the only public health facility in Tanzania offering comprehensive treatment for cancer patients from all regions in Tanzania. The institute serves as a teaching hospital for Muhimbili University of Health and Allied Sciences (MUHAS). The hospital has a 270-bed capacity and also offers outpatient clinics and advanced diagnostic services. ORCI receives an average of 10–15 new cancer patients every day and has 15 oncology specialists. More than 3000 new cases per year are recorded in the hospital-based registry.

### Study population eligibility

All chemotherapy naïve adult patients diagnosed to have solid tumors who were scheduled to receive a cisplatin-based regimen at ORCI were eligible. Patient’s history was noted to ensure that the recruited participants would complete the course of treatment.

### Inclusion and exclusion criteria

#### Inclusion criteria

1. Patients aged 18 years and above with confirmed diagnosis of a solid tumor.
2. Patients who had not received any prior cancer chemotherapy and were to receive their first course of cancer chemotherapy that included cisplatin (≥50 mg)/week.
3. Adequate renal function before the start of chemotherapy (baseline serum creatinine ≤115 μmol/L).
4. Adequate bone marrow function assessed by WBC ≥ 4.00 × 10^3^/μl, neutrophil count ≥2.00 × 10^3^/μl, lymphocyte count ≥0.8 × 10^3^/μl, platelets count ≥100.0 × 10^3^/μl, hemoglobin (Hb) ≥ 11.0 g/dl
5. Normal range of magnesium, potassium and calcium at baseline.
6. Signed informed consent

#### Exclusion criteria

1. Patients who were exposed to contrast media and those who had used potentially nephrotoxic drugs (non-steroidal anti-inflammatory drugs, aminoglycosides, amphotericin B, angiotensin-converting- enzyme inhibitors such as captopril and enalapril and angiotensin receptor blockers such as losartan) in the two weeks before recruitment were excluded.
2. Those with age above 70 yrs.
3. Pregnant women
4. HIV infected patients on antiretrovirals
5. Elevated serum creatinine (> 115 μmol/L) pretreatment
6. Patients with serum creatinine≥1.5 mg/dL and/or creatinine clearance< 50 ml/min

### A sampling of participants and follow up

All patients who received cisplatin-based chemotherapy were recruited into the study. Patients who were assigned to the group of cisplatin plus triple electrolytes supplementation were those who could buy the electrolytes as ORCI has not yet included the supplementation in the treatment package. Only the investigators were blinded but nurses and patients were not.

### Data collection

In this study Case Report Form (CRF) was used as a data collection tool to collect data of interest. CRF was designed to record all observations and other relevant data for each participant. Data from the source document (e.g. printed lab results) was entered into a case report form. A study clinician was responsible for filling up the case report form and filling were done at a clinic. To avoid introducing new treatment bias, the study followed all procedures done at the hospital.

### Social demographic information

Participant’s socio-demographic information of interest such as gender, age, weight, smoking status, alcohol intake was obtained from the patient file. These data were recorded on the CRF at baseline screening.

### Concomitant medications

The CRFs were designed to capture all concurrent therapies taken at baseline/screening in the CRF and reviewed at every visit. Dates of medications, dose, and frequency of administration were all captured.

### Medical history

Relevant medical history, including a history of current disease, and information regarding underlying diseases were recorded in the CRF at baseline.

### Adverse events information

Information regarding the occurrence of adverse events was captured throughout the study by taking the case history of patients during clinics. Duration (start and stop dates and times), severity/grade, outcome, treatment, and relation to study drug were assessed and recorded in the CRF.

### Laboratory measurements

The renal function test by measuring serum creatinine level was performed at baseline and on every visit until week 4. Measurements were performed at baseline screening and every visit. Serum measurements were performed on days 0, 7, 14, 21, and 28 just after chemotherapy. Serum electrolyte measurements were done at baseline to determine those who met the inclusion criteria. All laboratory investigations were performed at ORCI Laboratory Unit.

### Outcomes

#### Primary outcome

We used serum creatinine change from baseline after cisplatin administration as a primary outcome measure incidence of acute kidney injury. Nephrotoxicity was defined as serum creatinine elevation > 1.5 times that at baseline (grade ≥ 1) and was always measured on the 7th day of each clinic attendance just after administration of the next cisplatin dose.

#### Secondary outcome

Any other adverse event reported by patients during an interview or in the patient file.

### Participants’ treatment regimen

#### Preparation of hydration solutions

The hydration solutions were prepared by the hospital chemotherapy pharmacist in the chemotherapy mixing unit. Each electrolyte for supplementation was diluted in a separate liter of normal saline; as follows: Potassium chloride (KCl) (1.5 g), Magnesium sulfate (1 g) and calcium gluconate (1 g) and were each separately diluted in 1 L normal saline and added up to 3 L.

#### Delivery of treatments and interventions

The control group received 3 L normal saline while the treatment group received 3 L of normal saline in which the triple electrolyte supplementation was diluted. The solutions were prepared on the day of administration and the pharmacist ensured proper mixing by slowly shaking the bottle for approximately 10 times. The hydration fluid was administered for 6 h after which chemotherapy was given. The patient-specific cisplatin-based chemotherapy regimens varied from patient to patient as prescribed by the medical oncologist. Some patients received only cisplatin while others were prescribed a cisplatin-based regimen that contained two or three other cytotoxic drugs. The cisplatin injections were prepared as per the manufacturer’s instructions and administered as per prescription. The dose was diluted in 1 L of normal saline 30 min after hydration and administered by IV infusion over 90 min. All participants received a cisplatin dose ≥50 mg per week. Other medications given to participants included Granisetron 1 mg/ml injection, Dexamethasone 4 mg/ml injection, Ondansetron 8 mg tablet, and Dexamethasone 4 mg tablets. Besides hydration, participants were encouraged to drink a minimum of 500 ml of water daily, following the administration of cisplatin.

#### Data analysis

Data entry, cleaning, and analysis were done using Statistical Package for Social Science (SPSS) version 23.0. Continuous variables were expressed using measures of central tendency while categorical data such as serum creatinine values were expressed as log mean, frequencies, or proportions. A t-test was used to compare the mean Serum creatinine between the 2 groups from baseline to day 28. Z-test was used to compare the proportion of patients who had serum creatinine elevation 1.5 times baseline between the 2 groups. Univariate analysis was done using chi-square to determine the association between the different factors and AKI. Factors with *p*-values less than 0.2 in bivariate analysis were entered into a logistic regression to determine the association between nephrotoxicity and known associated factors noted on univariate analysis. Survival analysis was performed using Kaplan Meier to determine differences in time to an event using the log-rank test. A *P*-value of < 0.05 was considered statistically significant.

## Results

### Participants enrollment, allocation, and follow-up

**Fig. 1:**
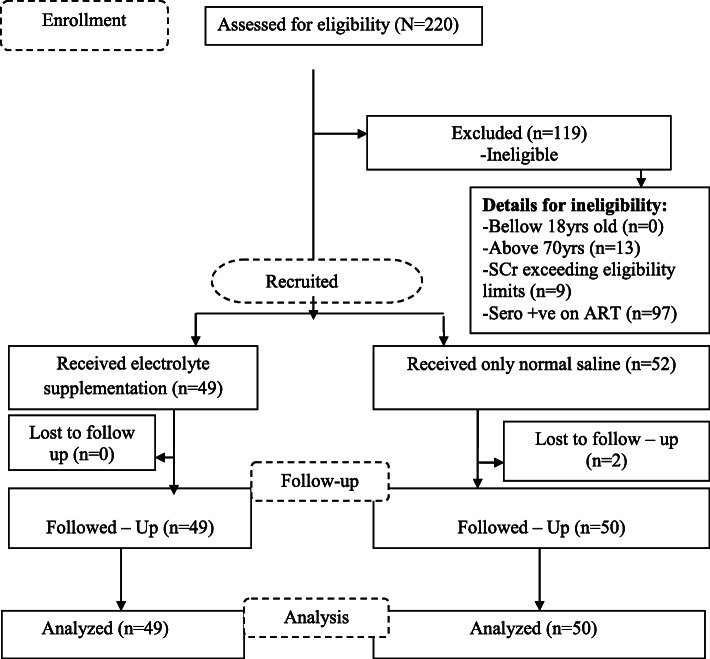
Patients recruitment process

The study took place between January 2019 and June 2019 and 220 patients were screened of which 119 were excluded, as they did not meet the inclusion criteria. Among them 13 were aged above 70 yrs., 97 patients were HIV positive on ART and 9 patients had elevated serum creatinine (> 115 μmol/L). One hundred and one patients (101) received either IV electrolyte supplementation in normal saline plus cisplatin (*n* = 49) or cisplatin in normal saline only (*n* = 52). Two patients in the group with no electrolytes (*n* = 52) did not show up on day 2 therefore were excluded from the final analysis. Ninety-nine participants who participated fully in this study were followed up for (4 weeks and their data were available for final analysis. Figure 1 summarizes the patient’s recruitment process.

### Participants baseline characteristics

A total of 99 participants were recruited and followed up, whereby49 patients (49.5%) received cisplatin+supplementation (treatment group) while 50 participants (51.5%) received cisplatin+normal saline. Most participants were females in both the electrolyte (*n* = 45, 91.8%) and sodium chloride group (*n* = 48, 96.0%). The majority of patients in both groups had age between 46 and 64 years. Most of the participants recorded BMI of 18.5–24.9 kg/m^2^ in both the electrolyte (*n* = 23, 46.9%) and in the normal saline group (*n* = 22, 44.0%) (*p* = 0.942). Most of the participants were neither cigarette smokers nor alcohol users (90%). Only 8.2% reported the use of traditional medicines (*p* = 0.168).

The most common malignancy was cervical cancer and accounted for 87.8% (43/49) and 86% (43/50) in the group that received electrolytes and normal saline respectively. Other malignancies were esophageal and oral cancers as described in Table 1.

**Table 1:** Participants Social Demographic and Clinical characteristics according to Electrolyte exposure

Characteristics	Study arms		*P*-value
	NaCl + Electrolyte n (%)	NaCl alone n (%)	
Sex			
Male	4 (8.2)	2 (4.0)	0.392
Female	45 (91.8)	48 (96.0)	
Age (years)			
30–45	18 (36.7)	7 (14.0)	0.114
46–64	21 (42.9)	35 (70)	
>65	10 (20.4)	8 (16.0)	
BMI (kg/m^2^)			
<18.5	6 (12.2)	5 (10.0)	0.942
18.5–24.9	23 (46.9)	22 (44.0)	
25–29.9	13 (26.5)	14 (28.0)	
≥ 30	7 (14.3)	9 (18.0)	
Smoking status			
Yes	2 (4.1)	1 (2.0)	0.552
No	47 (95.9)	49 (98.0)	
Alcohol use			
Yes	4 (8.2)	5 (10.0)	0.753
No	45 (91.8)	45 (90.0)	
Traditional medicine use			
Yes	4 (8.2)	1 (2.0)	0.168
No	45 (91.8)	49 (98.0)	
Co-morbidity			
Yes	6 (12.2)	6 (12.0)	0.640
No	43 (87.8)	44 (98.0)	
Type of cancer			
Cervical	43 (87.8)	45 (90.0)	0.590
Esophageal	4 (8.2)	1 (2.0)	
Oral	1 (2.0)	2 (4.0)	
Others	1 (2.0)	2 (4.0)	
Chemotherapy regimen use			
Cisplatin alone	47 (95.9)	46 (92.0)	0.418
Cisplatin contained regimen	2 (4.1)	4 (8.0)	
Cisplatin dose (mg)			
50–60	25 (51)	25 (50)	0.441
61–70	20 (40.8)	15 (30)	
71–80	2 (4.1)	7 (14)	
81–90	0	0	
>90	2 (4.1)	3 (6)	

### Comparison of serum creatinine level between the 2 arms

**Fig. 2:**
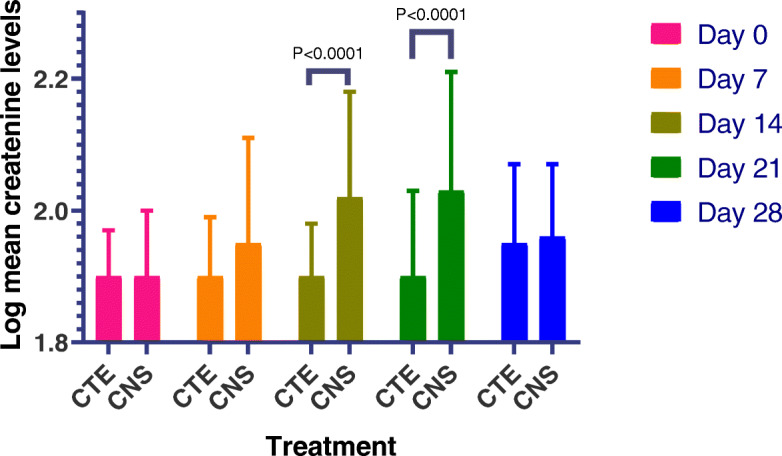
Serum creatinine levels between the two study groups (CTE: cisplatin + Triple electrolytes and CNS: cisplatin + normal saline)

The mean serum creatinine level at baseline and after cisplatin administration at different days was calculated and compared between the 2 groups. The baseline was comparable as well as day 28. However there was a significant statistical difference between the 2 groups on day 7,14 and day 21 with higher levels in the group that did not receive triple electrolytes supplementation with a more pronounced difference on day 21 (*p* < 0.0001). The results are summarized in Table 2 and Fig. 2.

**Table 2:** Comparison of mean Serum creatinine between the 2 arms from baseline to day 28

Day	Mean SCr Mean ± SD (mg/dl)		***P***-Value
	Electrolyte Supplementation Arm (n)	Non-electrolyte Arm (n)	
Day 0 (baseline)	0.9008 **±** 0.1430 (49)	0.8948 **±** 0.1960 (50)	0.8621
Day 7	0.9177 **±** 0.1941 (47)	1.075 **±** 0.3730 (44)	0.0126
Day 14	0.9902 **±** 0.1876 (44)	1.264 **±** 0.5493 (42)	0.0025
Day 21	0.8693 **±** 0.2385 (42)	1.315 **±** 0.6347 (41)	< 0.0001
Day 28	1.052 **±** 0.3602 (26)	1.060 **±** 0.2918 (25)	0.9305

The log means creatinine values were compared between the two groups and there was a statistically significant difference between the two arms on day 14 and 21 with higher levels in the group that did not receive electrolytes (*p* < 0.0001). However day 28 indicated no statistically significant difference in the log serum creatinine levels between the 2 groups although there were higher serum creatine in the normal saline group (Fig. 2).

### Proportion of patients with elevated serum creatinine level elevated> 1.5 times baseline in the two groups

The proportion of patients whose serum creatinine levels were elevated > 1.5 times baseline after cisplatin administration was calculated to determine those who experienced nephrotoxicity as per criterion mentioned previously. As illustrated in Table 3, there was a higher proportion of patients with serum levels elevation > 1.5 times baselines in all days in a group that did not receive triple electrolytes supplementation.

**Table 3:** Proportion of patients with serum creatinine elevation > 1.5 times that at baseline

Day	% Patients with serum creatinine elevation > 1.5		***P***-Value
	Electrolyte Supplementation Arm % (n)	Non-electrolyte Arm % (n)	
Day 7	6.1 (48)	30.2 (43)	0.0025
Day 14	8.9 (45)	36.6 (41)	0.002
Day 21	2.3 (43)	43.9 (41)	< 0.0001
Day 28	0 (27)	24 (25)	0.0068

### Cumulative incidence of nephrotoxicity

**Fig. 3:**
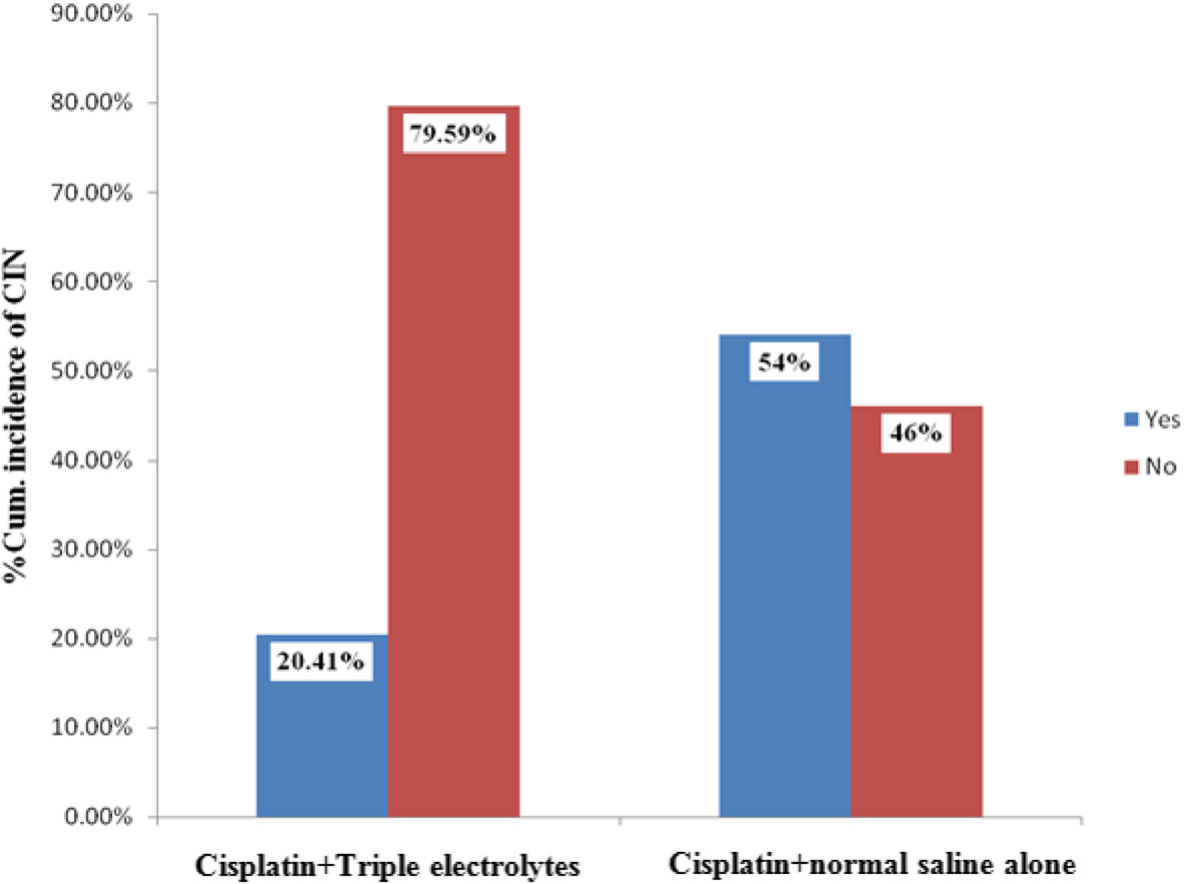
Cumulative incidence of nephrotoxicity serum creatinine elevation> 1.5 times baseline) in the cisplain+triple electrolytes and cisplatin+Normal saline alone group

Cumulative incidence risk of nephrotoxicity was 20% (10/49) in the participants who received electrolytes but was 54% (27/50), in those who did not receive electrolytes (Fig. 3). Participants who did not receive electrolytes supplementation were 2.6 times more likely to experience nephrotoxicity as compared to those who received [Relative Risks (RR); 2.6, 95%Confidence Interval (95%CI); 1.5–4.9, *p* < 0.0001].

### Assessment of other adverse events

The commonest adverse events noted in this study included vomiting and diarrhea. No difference in the occurrence of adverse effects was noted between the two groups.

### Time to experience nephrotoxicity after cisplatin administration

**Fig. 4:**
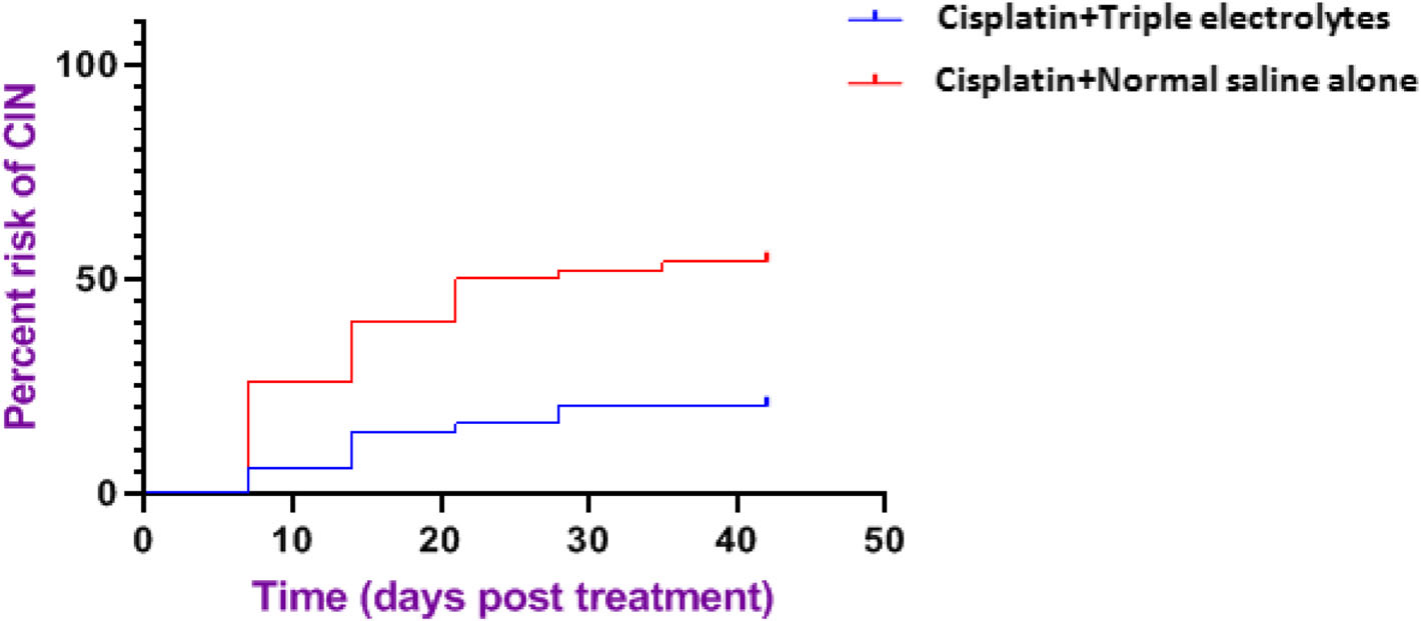
Kaplan Meier survival curves showing the comparison of time to nephrotoxicity (survival rate) between the two treatment groups

The time from the first dose of cisplatin-based chemotherapy to the development of cisplatin-induced nephrotoxicity (CIN) was noted to be 10 days in both groups. However, the risk of nephrotoxicity was significantly higher for participants who did not receive triple electrolytes supplementation [*p* = 0.0004; Hazard ratio (HR) 0.3149; 95% CI 0.165 to 0.6011], Fig. 4.

### Comparison of blood components between the two experimental arms

The blood components such as white blood cells, neutrophils, lymphocytes, blood platelets, and hemoglobin were compared between the two arms based on follow-up days. However, the difference between the two groups was not statistically significant for blood components for the entire follow up duration (*p* > 0.05).

### Predictors of cisplatin-induced nephrotoxicity

Different statistical tests were used to investigate the association between individual baseline characteristics (demographic and clinical characteristics) and the development of CIN. The bivariate analysis revealed that the baseline SrCr level was significantly associated with the development of CIN (*p* = 0.035). This association was also observed when comparing crreatinine clearance (CrCl) levels in the 2 groups (*p* = 0.028).

## Discussion

In this study, we compared the renal protective effect of triple electrolytes supplementation containing intravenous potassium chloride (1.5 g), magnesium sulfate (1 g) and calcium gluconate (1 g) among cancer patients who were undergoing cisplatin dose ≥50 mg/week with those who received cisplatin and normal saline alone. We found that a good proportion of patients in the group that received only normal saline had higher levels of serum creatinine and experienced nephrotoxicity after cisplatin dosing as opposed to the group that received triple electrolyte supplementation. Similarly, those who did not receive the triple electrolytes had a higher cumulative incidence risk of developing nephrotoxicity as opposed to those who received the triple electrolytes supplementation. Nephrotoxicity was established through a raised serum creatinine level (SCr) > 1.5 baselines. An increase in SCr is a marker of decreased renal CrCl and is an index of drug accumulation, which in turn provokes drug-induced nephrotoxicity.

In the current study, the difference in SCr elevation between those patients who were in cisplatin + triple electrolyte arm as compared to cisplatin plus normal saline alone was statistically significant. Additionally, patients in the triple electrolyte supplementation group showed no significant difference between pre and post-treatment SCr levels and between days (Fig. 2) indicating the role of electrolyte supplementation in ameliorating nephrotoxicity.

Several clinical studies have shown that cisplatin-based chemotherapy resulted in hypomagnesia, hypocalcaemia, hypophosphatemia, hypokalemia, and increased serum creatinine and blood urea nitrogen levels [10–13]. Therefore supplementing the extensively lost electrolytes will subsequently reduce renal tubular damage.

The exact mechanisms of cisplatin-induced nephrotoxicity have not been fully elucidated. However, several mechanisms for the development of nephrotoxicity have been proposed that include oxidative stress, DNA adducts, inflammation, mitochondrial dysfunction, and direct cytotoxicity to the tubular epithelial cells [13]. In 2016, Ruggiero et al. was able to show that nephrotoxicity associated with cisplatin is related to accumulation of metabolites in the renal proximal tubule cells of the kidneys, where about 90% of cisplatin undergoes urinary excretion [17]. Accumulation of these metabolites causes direct inflammation; the production of reactive oxygen species, which leads to oxidative cell damage; and cell death [17, 18]. Despite the proposed causes of nephrotoxicity, the mechanism through which the electrolytes are lost in patients taking cisplatin remains inconclusive.

Looking into the log mean serum creatinine values, a slight difference between the 2 arms at day 7 (*p* > 0.05) was observed. However at days 14 and 21, there was a significant statistical difference in serum creatinine elevation between the 2 arms (*p* = 0.0001). No significant difference was noted in the mean creatinine level on day 28 between the 2 groups. Lower levels of serum creatinine between days 14–21 in the triple electrolytes supplemented arm are attributed to the protective effect of supplemented electrolytes. In this study, the triple electrolyte supplementation could only be done up to week 3 after cisplatin initiation. The effect of stopping the electrolytes supplementation is well noted on day 28 where a reversed trend is observed in the supplemented arm. However, by day 28, none of the patients in the treatment arm had serum creatinine level higher than 1.5 times baseline compared to control group where 24% of the patients had their serum creatinine level higher than 1.5 times baseline (*p* = 0.0068) despite the means being comparable in Table 2.

The Kaplan Meier survival estimates analysis was conducted to compare the pattern of survival rates over time from the first day of cisplatin administration to the end of the follow-up period between the two groups. There was a higher risk of experiencing nephrotoxicity in the control group than the triple electrolyte supplementation group. The statistically significant difference found between the two survival curves indicates the beneficial effects of the triple electrolytes supplementation in extending the time to the development of nephrotoxicity. The survival curves in both treatment groups revealed that nephrotoxicity mostly occurred within 10 days following cisplatin administration with a higher risk in the arm that did not receive supplementation. Cisplatin is primarily eliminated unchanged via the kidney and has a terminal elimination half-life of up to10 days. It is only after 10 days post cisplatin administration when 50% of the drug remains in body explaining the reason for kidney injury to occur within 10 days post cisplatin administration in both groups [18–22]. The Kaplan Meier survival curve indicates there was a higher risk in the arm that did not receive supplementation. Since the drug is given weekly (not every after its half-life), the drug plasma concentration continues building up until it reaches a steady-state and this is the reason we see a higher incidence of nephrotoxicity in patients receiving cisplatin and normal saline alone compared to the supplemented group. Our findings are consistent with reports from other studies [9–13, 20].

Several risk factors accounting for nephrotoxicity during cisplatin chemotherapy were analyzed and revealed that only baseline SrCr level was significantly associated with the development of nephrotoxicity and this is in line with findings that have been reported previously [8]. With high level serum creatinine at baseline, the kidney is prone to injury once exposed to nephrotoxic agents such as cisplatin.

To our knowledge, this is the first study to report the renal protective effect of the triple electrolyte supplementation (magnesium, potassium, and calcium) given just before the administration of cisplatin-based chemotherapy at OCRI in Tanzania. The data obtained from this study provides evidence to support the remarkable renal protective effect by supplementing three electrolytes during the use of cisplatin in the treatment of cancers. Our finding warrants further investigations with a larger sample size before the triple electrolyte supplementation protocol is adopted nationwide.

However, in this study only complete blood count and serum creatinine could be measured hence we missed some of the important information that could be obtained from other tests. In ddition, the  levels of electrolyte could be measured only at baseline but no additional measurements were made during follow up after subsequent cisplatin administrations. Besides only a small number of patients could be recruited to the end of the study  period underpinning the need of conducting the study in a bigger population with revised inclusion and exclusion criteria.

## Conclusions

Electrolytes supplementation decreases the risk of nephrotoxicity after chemotherapy with cisplatin. A randomized controlled trial with a larger sample size is recommended to evaluate the robustness of these findings.

## Data Availability

All relevant data generated and analyzed during this study are available on reasonable request. The corresponding author should be contacted to avail data after obtaining permission from the director of research and publications at Mubimbili University of Health and Allied Sciences.

## Abbreviations

AKI: Acute Kidney Injury
BMI: Body Mass Index
BUN: Blood Urea Nitrogen
CIN: Cisplatin-induced Nephrotoxicity
CrCl: Creatinine Clearance
CRF: Case Report Form
ORCI: Ocean Road Cancer Institute
SCr: Serum creatinine
